# Serum pentosidine levels are associated with reduced eGFR, including in individuals with preserved renal function: a cross-sectional analysis from the Fukuoka Epidemiological Study of Atherosclerosis

**DOI:** 10.1186/s12882-026-05022-9

**Published:** 2026-05-12

**Authors:** Kenji Ito, Koji Takahashi, Kazuhiro Tada, Toshiki Maeda, Yukiko Shinohara, Yori Inoue, Atsushi Satoh, Makiko Abe, Tetsuhiko Yasuno, Hisatomi Arima, Kosuke Masutani

**Affiliations:** 1 Division of Nephrology and Rheumatology, Department of Internal Medicine, Faculty of Medicine, Fukuoka University, 7-45-1 Nanakuma, Jonan-ku, Fukuoka, 814-0180 Japan; 2 Department of Preventive Medicine and Public Health, Faculty of Medicine, Fukuoka University, Fukuoka, Japan; 3 Laboratory of Epidemiology and Prevention, Kobe Pharmaceutical University, Kobe, Japan

**Keywords:** Advanced glycation end products, Pentosidine, Chronic kidney disease

## Abstract

**Background:**

Advanced glycation end products, particularly pentosidine, are implicated in age-related diseases and are influenced by renal function and glycemic status. Although elevated pentosidine levels are well documented in individuals with chronic kidney disease (CKD) stage G3b or higher, their association with early reductions in estimated glomerular filtration rate (eGFR), including in individuals with preserved renal function, remains incompletely understood.

**Methods:**

We conducted a cross-sectional analysis of 813 Japanese adults from the Fukuoka Epidemiological Study of Atherosclerosis who underwent routine health checkups. Serum pentosidine and Nε-(carboxymethyl)lysine (CML) levels were measured and analyzed in relation to eGFR categories and glycemic markers.

**Results:**

Serum pentosidine levels were significantly associated with early reductions in eGFR, even among individuals with values including individuals with preserved renal function (P for trend < 0.01), whereas serum CML levels remained largely unchanged. In multivariable logistic regression analysis, lower eGFR category was significantly associated with elevated serum pentosidine levels (odds ratio, 1.94; 95% confidence interval, 1.40–2.70) after adjustment for covariates. In contrast, no significant association was observed between HbA1c-defined glycemic status and serum pentosidine levels. Triglycerides, high-sensitivity C-reactive protein (hs-CRP), and obesity (body mass index ≥ 25 kg/m²) were inversely associated with serum pentosidine levels.

**Conclusions:**

Lower eGFR, including in individuals with preserved renal function, was associated with higher serum pentosidine levels in this general population. These findings suggest that subtle reductions in renal function, even in individuals with preserved renal function, are associated with higher serum pentosidine levels, which may reflect a combination of cumulative oxidative stress and altered renal handling.

**Supplementary Information:**

The online version contains supplementary material available at 10.1186/s12882-026-05022-9.

## Background

Advanced glycation end products (AGEs) comprise a diverse group of bioactive compounds formed through the nonenzymatic glycation and oxidation of proteins, lipids, and nucleic acids [1]. Their accumulation is accelerated by chronic hyperglycemia, oxidative stress, and impaired metabolic clearance, and they are increasingly recognized as contributors to the pathophysiology of age-related diseases, including atherosclerosis, diabetic complications, and conditions associated with impaired renal function [2,3]. AGEs exert their deleterious effects through structural protein modifications, crosslinking of extracellular matrix components, and activation of proinflammatory and profibrotic signaling pathways via receptors such as the receptor for AGEs [4,5].

Among the various AGE species, pentosidine—a fluorescent crosslinking AGE formed through the glycoxidation of lysine and arginine residues in the presence of pentoses—is considered a marker of systemic AGE burden [6]. Its accumulation has been documented in patients with diabetes and microvascular and/or macrovascular complications [7]. Elevated pentosidine levels have also been associated with cardiovascular disease, sarcopenia, and frailty [8,9]. In addition to hyperglycemia, renal function is considered an important determinant of circulating AGE levels, as reduced renal clearance and enhanced oxidative and inflammatory stress may promote endogenous AGE production through mechanisms that are at least partly independent of glycemic exposure [10].

Serum pentosidine levels have been shown to be markedly elevated in patients with CKD stage G3b or higher, including those undergoing dialysis, reflecting impaired renal elimination as well as systemic metabolic disturbances [10,11]. However, little is known about the influence of earlier reductions in eGFR, including in individuals with preserved renal function, on circulating pentosidine levels in a general population setting. Notably, recent data have suggested that even modest reductions in estimated glomerular filtration rate (eGFR), including values within or near the conventionally normal range, may be accompanied by altered AGE handling and possibly subtle oxidative stress [12]. Such early AGE accumulation may reflect pathophysiological processes related to renal aging, potentially involving altered renal handling, subtle oxidative stress, and early tissue vulnerability, even before overt CKD becomes clinically apparent. Given that AGEs contribute to progressive organ damage, understanding their behavior during these early phases of renal functional decline is of considerable clinical relevance.

To date, the relationship between renal function, glycemic status, and serum AGE levels—particularly pentosidine—remains incompletely understood in the general population. Moreover, the heterogeneity of AGE species and their distinct biochemical pathways supports an approach that evaluates specific AGEs rather than relying solely on composite AGE measurements. For instance, Nε-(carboxymethyl)lysine (CML), another AGE formed primarily via the oxidation of Amadori products or lipid peroxidation, may exhibit different kinetics and renal handling compared with pentosidine [13].

In this study, we aimed to investigate the association between serum pentosidine levels and early reductions in eGFR, including individuals with preserved renal function, in a Japanese population undergoing routine health checkups. We also aimed to examine the relationship between pentosidine and glycemic status, and to compare the behavior of pentosidine with that of another AGE species, CML, to better understand the relative contributions of renal function and glycemic status to circulating pentosidine levels.

## Methods

### Study design and participants

The Fukuoka Epidemiological Study of Atherosclerosis (FESTA) project is a cross-sectional epidemiological study aimed at identifying both risk factors for and protective factors against atherosclerotic diseases. This study initially included 866 consecutive participants aged ≥ 40 years who underwent routine community-based health checkups between 2019 and 2022 in specific regions of Fukuoka Prefecture (Jonan Ward in Fukuoka City, and all of Nakagawa City), Japan. Participants were enrolled without specific selection criteria other than attendance at the health checkups. Data collection, including clinical measurements and biospecimen sampling, was performed at the time of the health checkups. All participants provided informed consent after receiving an explanation of the study before enrollment. We excluded 24 individuals who withdrew their consent and 17 for whom AGE measurements could not be performed because of insufficient surplus serum samples. Detailed information on medication use, smoking history, and past medical history, including hypertension and diabetes treatment, was not available in the health checkup dataset and was therefore not included in the present analysis.

Estimated glomerular filtration rate (eGFR) was primarily calculated using the equation established by the Japanese Society of Nephrology [14] To focus on early reductions in renal function, participants with eGFR < 45 mL/min/1.73 m² were excluded (*n* = 12). This threshold was used as a study-specific exclusion criterion to remove individuals with CKD stage G3b or higher, in whom impaired AGE clearance has been well established [11]. Accordingly, the analytic cohort consisted of individuals with preserved renal function or mild renal impairment (eGFR ≥ 45 mL/min/1.73 m²). Consequently, a total of 813 participants were included in the final analysis. In addition, sensitivity analyses were performed by recalculating eGFR using the Chronic Kidney Disease Epidemiology Collaboration (CKD-EPI) equation and by restricting the study population to participants with eGFR ≥ 60 mL/min/1.73 m² [15, 16]. This study was approved by the Fukuoka University Clinical Research and Ethics Center (approval no.: 2018M078).

### Health check-up data acquisition

Height and weight were measured without jackets or shoes and body mass index (BMI) was calculated as weight (kg) divided by height squared (m²). Blood pressure was measured in the right upper arm using mercury, automated, or aneroid sphygmomanometers equipped with appropriately sized cuffs. The measurements were performed by trained staff, with the participants seated comfortably after resting for at least five minutes. Fasting blood and urine samples were collected. Serum creatinine levels were measured using an enzymatic method, and eGFR was calculated using the following equation established by the Japanese Society of Nephrology: eGFR (mL/min/1.73 m²) = 194 × serum creatinine^(–1.094)^ × age^(–0.287)^ × 0.739 (if female) [14]. This equation is widely used and validated in Japanese populations.

### Assessment of serum AGEs

Surplus serum collected during health check-ups was frozen at − 80 °C after initial testing. Serum pentosidine and CML concentrations, the primary variables of interest in this study, were measured as contract assays by Fushimi Pharmaceutical Co., Ltd. (Marugame, Japan). Serum pentosidine was quantified using high-performance liquid chromatography with fluorescence detection (inter-assay coefficient of variation, 5.4%), whereas serum CML was measured using an enzyme-linked immunosorbent assay employing antibodies from the same manufacturer (inter-assay coefficient of variation, 5.6%). Participants were divided into high and low pentosidine groups according to the median serum pentosidine level (high: *n* = 407; low: *n* = 406).

### Supplementary assessments

Surplus urine samples collected during health check-ups were frozen at − 80 °C after initial testing for supplementary urinary assessment. Serum high-sensitivity C-reactive protein (hs-CRP) and urinary albumin, sodium, and potassium levels were measured by the Fukuoka Public Health Promotion Organization (Fukuoka, Japan). Serum hs-CRP was measured using a latex-enhanced immunoturbidimetric assay with reagents from Nitto Boseki Co., Ltd. (Koriyama, Japan). Urinary albumin was measured using an immunoturbidimetric assay, and urinary sodium and potassium concentrations were determined using ion-selective electrode methods with reagents from Nitto Boseki Co., Ltd for sodium, and A&T Corporation (Fujisawa, Japan) for potassium, respectively, to calculate the urinary albumin-to-creatinine ratio (ACR), estimated salt intake, and estimated potassium intake.

### Statistical analysis

SPSS Statistics version 26 (IBM Corp., Armonk, NY, USA) was used to perform all statistical analyses in this study. For comparisons between the two groups, normally distributed continuous variables were expressed as means (standard deviations) and analyzed using the Student’s t-test. Non-normally distributed continuous variables were presented as medians (interquartile ranges), and analyzed using the Mann-Whitney U test. Categorical parameters were expressed as frequencies and analyzed using the Chi-squared test. Trend analysis was conducted using the Jonckheere-Terpstra test. Multivariable logistic regression analysis was performed to examine the factors that influenced elevated serum pentosidine levels. In addition, multivariable linear regression analysis was performed with log-transformed serum pentosidine as a dependent variable. eGFR and HbA1c were analyzed both as a categorical variable and as a continuous variable (per 10 mL/min/1.73 m² decrease and per 1% increase). Sensitivity analyses were conducted by recalculating eGFR using the Chronic Kidney Disease Epidemiology Collaboration (CKD-EPI) Eqs. [15, 16] and by restricting the study population to participants with eGFR ≥ 60 mL/min/1.73 m². Covariates included in the multivariable models were selected based on clinical relevance, including age, sex, BMI, systolic blood pressure, glycemic status, lipid profile, and inflammatory markers. Analyses were conducted using available data for each variable, and no imputation was performed for missing data. All reported P-values were two-tailed, and statistical significance was set at *P* < 0.05.

### Use of artificial intelligence

An AI-assisted language model (ChatGPT, OpenAI, San Francisco, CA, USA) was used to improve the clarity and consistency of the English language; the authors take full responsibility for the content of the manuscript.

This study was reported in accordance with the Strengthening the Reporting of Observational Studies in Epidemiology (STROBE) guidelines [17]. The STROBE checklist [18] was used during manuscript preparation and is provided as Supplementary material.

## Results

### Participant characteristics

The clinical characteristics of the study participants are summarized in Table 1. Their mean age was 63.9 (9.4) years, 45.6% were male, and 61.1% were aged ≥ 65 years. The distributions of their eGFR categories were: G1 (6.8%), G2 (75.3%), and G3a (17.9%), indicating that the study population predominantly consisted of individuals with preserved or mildly reduced renal function. Their mean fasting glucose and glycated hemoglobin (HbA1c) levels were 97.7 (17.3) mg/dL and 5.68 (0.58)%, respectively, and 6.8% of the participants had HbA1c levels ≥ 6.5%. Baseline characteristics based on eGFR calculated using the CKD-EPI equation are shown in Supplementary Table 1.

**Table 1:** Baseline characteristics of the study participants according to serum pentosidine levels

	Total (*N* = 813)	High Pentosidine (*N* = 407)	Low Pentosidine (*N* = 406)	*P* value
Age (years)	63.9 (9.4)	66.1 (7.9)	61.7 (10.3)	< 0.01
Male, n (%)	371 (45.6)	178 (43.7)	193 (47.5)	0.29
BMI (kg/m²)	22.7 (3.2)	22.1 (3.0)	23.4 (3.3)	< 0.01
Waist circumference (cm)	82.7 (9.1)	80.9 (8.6)	84.7 (9.2)	< 0.01
Systolic BP (mmHg)	127.1 (17.0)	126.4 (17.1)	127.9 (16.9)	0.22
Diastolic BP (mmHg)	74.3 (11.0)	73.5 (10.5)	75.2 (11.4)	0.022
eGFR (mL/min/1.73 m²)	71.4 (12.8)	68.8 (11.5)	74.1 (13.5)	< 0.01
eGFR category, n (%)				< 0.01
eGFR ≥ 90 mL/min/1.73 m² (%)	55 (6.8)	17 (4.2)	38 (9.3)	
eGFR 60–89 mL/min/1.73 m² (%)	612 (75.3)	293 (72.0)	319 (78.6)	
eGFR < 60 mL/min/1.73 m² (%)	146 (17.9)	97 (23.8)	49 (12.1)	
Urinary ACR (mg/g creatinine)	3.61 (2.01–6.90)	3.87 (2.19–7.45)	3.41 (1.79–6.53)	0.66
Estimated salt intake (g/day)	9.0 (2.0)	9.0 (2.0)	8.9 (2.0)	0.29
Estimated potassium intake (mg/day)	3247 (784)	3275 (781)	3218 (787)	0.29
High-sensitivity CRP (mg/dL)	0.040 (0.019–0.091)	0.031 (0.015–0.073)	0.049 (0.024–0.102)	< 0.01
Fasting glucose (mg/dL)	97.7 (17.3)	97.0 (14.5)	98.5 (19.7)	0.25
HbA1c (NGSP, %)	5.68 (0.58)	5.68 (0.53)	5.68 (0.63)	0.96
TG (mg/dL)	96 (71–136)	84 (66–118.5)	107 (77–156)	< 0.01
LDL-C (mg/dL)	134.0 (33.2)	133.1 (33.9)	135.0 (32.5)	0.40
HDL-C (mg/dL)	68.7 (17.7)	72.6 (18.0)	64.8 (16.5)	< 0.01
Pentosidine (µg/mL)	0.018 (0.014–0.022)	0.026 (0.023–0.028)	0.016 (0.013–0.019)	< 0.01
CML (µg/mL)	4.02 (0.54)	4.03 (0.52)	4.01 (0.55)	0.74

Values are presented as mean (standard deviation), median (interquartile range), or number (percentage), as appropriate

Comparisons between groups were performed using the Student’s t-test for normally distributed continuous variables, the Mann–Whitney U test for non-normally distributed continuous variables, and the chi-squared test for categorical variables

eGFR was calculated using the equation established by the Japanese Society of Nephrology

Abbreviations: BMI, body mass index; BP, blood pressure; eGFR, estimated glomerular filtration rate; Cr, creatinine; ACR, albumin-to-Cr ratio; CRP, C-reactive protein; HbA1c, hemoglobin A1c; NGSP, National Glycohemoglobin Standardization Program; TG, triglycerides; LDL-C, low-density lipoprotein cholesterol; HDL-C, high-density lipoprotein cholesterol; CML, Nε-(carboxymethyl)lysine

### Association between serum AGE levels and renal function

**Fig. 1:**
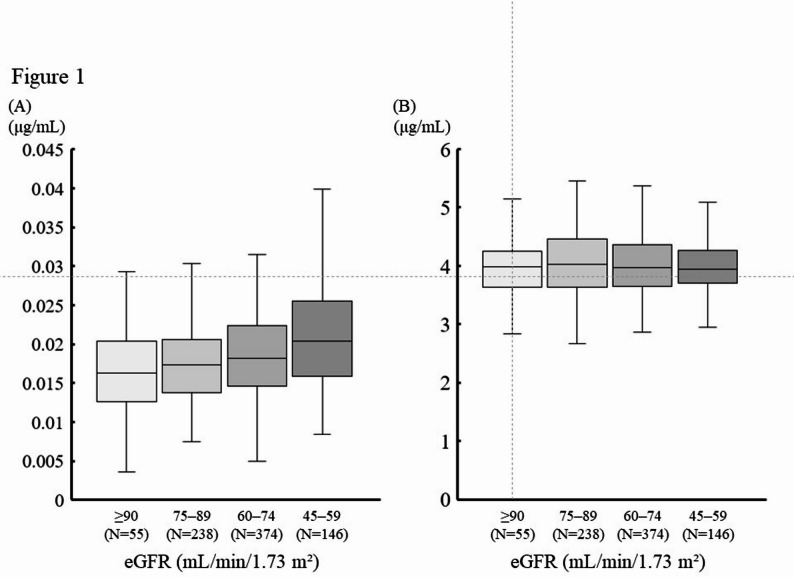
Serum pentosidine and N^ε-(carboxymethyl)lysine (CML) levels according to estimated glomerular filtration rate (eGFR) categories. eGFR was grouped into approximately 15 mL/min/1.73 m² intervals for descriptive visualization of trends across the preserved and mildly reduced renal function range. (**A**) Serum pentosidine levels showed a significant increasing trend with declining eGFR, including among individuals with preserved renal function (P for trend < 0.01). (**B**) Serum CML levels did not significantly change across eGFR categories (P for trend = 0.60) Values are presented as box-and-whisker plots

Figure 1 illustrates the relationship among the measured concentrations of pentosidine, CML, and eGFR in the participants. The study population predominantly consisted of individuals with preserved or mildly reduced renal function, with approximately 80% having an eGFR of ≥ 60 mL/min/1.73 m². Serum pentosidine levels increased significantly with declining eGFR across categories, including among individuals with preserved renal function (P for trend < 0.01). By contrast, serum CML levels remained largely unaffected across eGFR categories (P for trend = 0.60), indicating a differential association between specific AGE species and renal function.

### Comparison between high and low serum pentosidine

The clinical characteristics of the participants were compared between the high and low serum pentosidine groups, classified according to the median serum pentosidine level (Table 1). Both age and high-density lipoprotein cholesterol (HDL-C) levels were significantly higher in the high pentosidine group (age: 66.1 (7.9) years vs. 61.7 (10.3) years, *P* < 0.01; HDL-C: 72.6 (18.0) mg/dL vs. 64.8 (16.5) mg/dL, *P* < 0.01). Similar trends were observed when eGFR was calculated using the CKD-EPI equation (Supplementary Table 1).

By contrast, BMI, waist circumference, and eGFR were significantly lower in the high pentosidine group, and hs-CRP and triglyceride (TG) levels were also lower in this group, suggesting a distinct metabolic profile associated with higher pentosidine levels. Although hyperglycemia is known to influence pentosidine production, no significant differences in fasting blood glucose or HbA1c levels were observed, suggesting that glycemic status may not have been a major determinant in this population.

### Logistic regression analysis of factors associated with high serum pentosidine levels

A multivariable logistic regression model was used to identify clinical variables associated with elevated serum pentosidine levels (Table 2). The following variables were included as predictors in the model: age (per 10-year increase), sex (male), obesity (BMI ≥ 25 kg/m²), eGFR category (per category decrease), high urinary ACR (> 75th percentile), high hs-CRP (> 75th percentile), systolic blood pressure (per 10 mmHg increase), high HbA1c (≥ 6.5%), high TG (≥ 150 mg/dL), and low HDL-C (< 40 mg/dL).

Age and lower eGFR category were significantly associated with elevated serum pentosidine levels, with eGFR category showing an independent association (odds ratio [OR], 1.94; 95% confidence interval [CI], 1.40–2.70; *P* < 0.01). High HbA1c was not identified as a significant predictor of elevated serum pentosidine.

High hs-CRP and TG levels, as well as obesity, were inversely associated with serum pentosidine levels, with the following ORs: obesity, OR 0.57 (95% CI, 0.39–0.85; *P* < 0.01); high hs-CRP, OR 0.69 (95% CI, 0.48–0.98; *P* = 0.04); and high TG, OR 0.50 (95% CI, 0.34–0.73; *P* < 0.01), suggesting a distinct metabolic and inflammatory profile associated with higher pentosidine levels.

These findings were generally consistent in sensitivity analyses using the CKD-EPI equation (Supplementary Table 2), whereas in analyses restricted to participants with eGFR ≥ 60 mL/min/1.73 m² (Supplementary Table 3), the association was attenuated in the logistic regression model.

In multivariable linear regression analysis with log-transformed serum pentosidine as the dependent variable (Table 2), lower eGFR was significantly associated with higher pentosidine levels when analyzed as a continuous variable (per 10 mL/min/1.73 m² decrease; B = 0.06, 95% CI 0.03 to 0.08; *P* < 0.01). Consistent with the logistic regression analysis, obesity, high hs-CRP, and high TG levels were inversely associated with pentosidine. HbA1c was not significantly associated with pentosidine levels when analyzed as a continuous variable. These findings were generally consistent in the sensitivity analyses (Supplementary Tables 2 and 3). In analyses restricted to participants with eGFR ≥ 60 mL/min/1.73 m², the association remained significant in the linear regression model, although it was attenuated in the logistic regression model.

**Table 2:** Factors associated with serum pentosidine levels: logistic and linear regression analyses

	Univariable logistic regression			Multivariable logistic regression			Multivariable linear regression		
	OR	95% CI	*P* value	OR	95% CI	*P* value	B	95% CI	*P* value
Age (per 10 years)	1.58	1.36–1.83	< 0.01	1.50	1.27–1.77	< 0.01	0.07	0.04 to 0.10	< 0.01
Sex (male)	0.86	0.65–1.13	0.28	1.15	0.84–1.56	0.38	0.03	-0.03 to 0.08	0.30
Obesity (BMI ≥ 25 kg/m²)	0.48	0.34–0.69	< 0.01	0.57	0.39–0.85	< 0.01	-0.09	-0.16 to -0.03	< 0.01
eGFR category (per category decrease)	2.12	1.57–2.87	< 0.01	1.94	1.40–2.70	< 0.01	—	—	—
eGFR (per 10 mL/min/1.73 m² decrease)	—	—	—	—	—	—	-0.06	-0.08 to -0.03	< 0.01
Urinary ACR (> 75th percentile)	1.18	0.86–1.63	0.30	1.10	0.78–1.56	0.57	0.01	-0.05 to 0.07	0.66
High-sensitivity CRP (> 75th percentile)	0.66	0.48–0.91	0.01	0.69	0.48–0.98	0.04	-0.07	-0.13 to -0.01	0.03
Systolic blood pressure (per 10 mmHg increase)	0.96	0.89–1.04	0.36	0.95	0.87–1.04	0.23	-0.02	-0.03 to -0.002	0.04
HbA1c (NGSP) ≥ 6.5%	0.88	0.49–1.56	0.65	0.98	0.52–1.86	0.96	—	—	—
HbA1c (NGSP) (per 1% increase)	—	—	—	—	—	—	-0.01	-0.05 to 0.04	0.84
TG > 150 mg/dL	0.42	0.29–0.60	< 0.01	0.50	0.34–0.73	< 0.01	-0.10	-0.16 to -0.03	< 0.01
HDL-C < 40 mg/dL	2.19	0.88–5.44	0.09	1.18	0.42–3.29	0.76	0.09	-0.08 to 0.25	0.30

Odds ratios (ORs) and 95% confidence intervals (CIs) were estimated using logistic regression models. Regression coefficients (B) and 95% CIs were estimated using linear regression models

Age was modeled per 10-year increase. eGFR category was treated as an ordinal variable (per category decrease), and eGFR was additionally analyzed as a continuous variable (per 10 mL/min/1.73 m² decrease). Obesity was defined as body mass index ≥ 25 kg/m². High urinary ACR and high hs-CRP were defined as values above the 75th percentile. High triglyceride levels were defined as ≥ 150 mg/dL, low HDL-C as < 40 mg/dL, and high HbA1c as ≥ 6.5%

Abbreviations: OR, odds ratio; CI, confidence interval; BMI, body mass index; CKD, chronic kidney disease; eGFR, estimated glomerular filtration rate; ACR, albumin-to-creatinine ratio; CRP, C-reactive protein; HbA1c, hemoglobin A1c; NGSP, National Glycohemoglobin Standardization Program; TG, triglycerides; HDL-C, high-density lipoprotein cholesterol

## Discussion

In this study, using data from the FESTA cohort—a health checkup–based population with largely preserved renal function—we demonstrated that serum pentosidine levels were associated with lower eGFR, inclusing among individuals with preserved renal function. In multivariable analysis, high HbA1c was not significantly associated with elevated serum pentosidine levels, whereas lower eGFR remained independently associated. These findings suggest that serum pentosidine levels may be influenced by factors beyond glycemic status and may reflect reduced renal handling, with possible additional contributions from oxidative processes.

Mechanisms related to renal handling should first be considered when interpreting the association between serum pentosidine levels and eGFR. Pentosidine is a small molecule that can be filtered at the glomerulus, and reduced renal function may lead to its accumulation in the circulation [11]. Indeed, the attenuation of the association between eGFR category and high pentosidine levels in analyses restricted to participants with eGFR ≥ 60 mL/min/1.73 m² supports the contribution of renal clearance to circulating pentosidine levels.

However, the differential responses of pentosidine and CML to early reductions in eGFR observed in this study suggest that mechanisms beyond simple renal clearance may also be involved. Although both are classified as AGEs, pentosidine is formed through glycoxidation reactions under oxidative conditions, and its production is influenced by oxidative stress and metal-catalyzed oxidation [19]. In contrast, CML is primarily generated through the oxidation of Amadori products or lipid peroxidation–derived aldehydes [20]. The difference in biosynthetic pathways may partly explain why pentosidine appears more sensitive to early renal functional changes, potentially through mechanisms involving altered AGE handling and subtle oxidative processes, even among individuals with preserved renal function.

In addition, differences in renal handling between these AGEs may contribute to their distinct associations with eGFR. While both circulate predominantly in a protein-bound form, free pentosidine is thought to be filtered and subsequently reabsorbed and degraded in the renal tubules, resulting in limited urinary excretion.^21^ In contrast, a larger proportion of free CML is excreted into the urine without tubular reabsorption [22]. Given that circulating free fractions of these AGEs are relatively small, especially in individuals with preserved renal function, the contribution of direct renal excretion alone may be insufficient to fully explain the observed differences. Extrarenal pathways, including intestinal metabolism and gut microbiota, may contribute to AGE clearance, particularly for CML [23], which may partly explain its weaker association with renal function in the present study.

Elevated pentosidine levels have been associated with adverse outcomes in various clinical settings. In patients with diabetes, high serum pentosidine concentrations are associated with increased risks of microvascular complications such as retinopathy and nephropathy [24], as well as macrovascular disease and cardiovascular mortality [8]. Moreover, recent studies have demonstrated significant associations between serum pentosidine levels and sarcopenia, suggesting broader effects on frailty and functional decline in older adults [9]. In patients with established CKD, pentosidine has been proposed as a surrogate marker of oxidative stress and inflammation—two major contributors to cardiovascular and all-cause mortality in this population [11,25].

Although the absolute differences in pentosidine levels observed across eGFR categories in the present study were relatively small, the consistent association with renal function may indicate that even subtle elevations reflect early biological changes. These findings raise the possibility that serum pentosidine may reflect early alterations in renal physiology, although longitudinal studies are required to determine its predictive value for future renal and cardiovascular outcomes.

In the present study, serum pentosidine levels were inversely associated with metabolic and inflammatory factors, including triglycerides, hs-CRP, and obesity, even after multivariable adjustment. Although oxidative stress and inflammation are generally considered to promote AGE formation, several factors may explain these seemingly paradoxical findings.

First, markers such as hs-CRP reflect short-term inflammatory activity, whereas pentosidine accumulates over longer periods and may not parallel transient changes in inflammation. Consistent with this interpretation, previous studies have reported only weak correlations between pentosidine and inflammatory markers, suggesting a limited contribution of acute inflammation [25]. Second, oxidative stress–related AGE accumulation has been reported to be pronounced in individuals with sarcopenia or undernutrition [9,26,27]. Because the present study population consisted of older adults and included individuals with lower BMI and triglyceride levels, these population characteristics may have contributed to the observed inverse associations. These findings should therefore not be interpreted as indicating protective effects of adverse metabolic profiles, but rather as reflecting distinct pathophysiological processes related to aging, oxidative stress, and renal handling.

This study has several limitations. First, its cross-sectional design precludes causal inference. Although an association between serum pentosidine levels and eGFR was observed, longitudinal studies are required to clarify the temporal relationship between early renal functional decline and AGE accumulation. Second, the study population consisted of individuals undergoing health checkups in specific regions of Japan, which may limit generalizability to other populations. Third, several relevant variables were not available or not assessed. Key biochemical measurements, including serum albumin, urinary AGE excretion, and glycated albumin, were not obtained, limiting assessment of nutritional status, renal handling mechanisms, and short-term glycemic variability. In addition, important potential confounders, such as smoking status, history of hypertension, and comorbid conditions including inflammatory diseases, malignancy, and frailty-related conditions, were not adequately captured. Furthermore, factors related to AGE metabolism and accumulation, including dietary AGE intake, antioxidant status, medication use (e.g., statins, angiotensin receptor blockers, and glucose-lowering medications), and genetic determinants, were not assessed. Nevertheless, some potential sources of bias may have been reduced by the use of consecutive recruitment and standardized data collection procedures.

Despite these limitations, this study has notable strengths. Whereas most previous studies have focused on populations with advanced CKD,^11,25^ the present analysis was conducted in a well-characterized general health checkup population with largely preserved renal function, enabling detection of subtle biochemical changes associated with early reductions in eGFR. Furthermore, concurrent measurement of two distinct AGE species allowed comparative assessment of their differential associations with renal function. By accounting for multiple metabolic and inflammatory factors, our analyses provided insight into the relative contributions of renal function and glycemic status to serum pentosidine levels.

## Conclusions

This study showed that serum pentosidine levels in the general Japanese population were associated with lower eGFR, including in individuals with preserved renal function, whereas CML levels remained largely unchanged. Multivariable analyses revealed that pentosidine was significantly associated with renal function, whereas no significant association was observed with HbA1c. These findings extend previous observations from patient-based studies to a health checkup population and suggest that serum pentosidine may reflect a combination of cumulative oxidative stress and altered renal handling, even in individuals with preserved renal function. Further longitudinal studies are warranted to clarify the clinical and prognostic implications of these observations.

## Electronic supplementary material

Below is the link to the electronic supplementary material.

Supplementary Material 1

## Data Availability

The datasets generated and/or analyzed during the current study are not publicly available due to privacy and ethical restrictions, but are available from the corresponding author on reasonable request.

## Abbreviations

AGEs: Advanced glycation end products
ACR: Albumin-to-creatinine ratio
BMI: Body mass index
BP: Blood pressure
CI: Confidence interval
CKD: Chronic kidney disease
CKD-EPI: Chronic Kidney Disease Epidemiology Collaboration
CML: Nε-(carboxymethyl)lysine
eGFR: Estimated glomerular filtration rate
FESTA: Fukuoka Epidemiological Study of Atherosclerosis
HbA1c: Hemoglobin A1c
HDL-C: High-density lipoprotein cholesterol
hs-CRP: High-sensitivity C-reactive protein
OR: Odds ratio
TG: Triglyceride
