# Supplementary material for: Serum pentosidine levels are associated with reduced eGFR, including in individuals with preserved renal function: a cross-sectional analysis from the Fukuoka Epidemiological Study of Atherosclerosis
Source: BMC Nephrol. 2026 May 12;27:408. doi: 10.1186/s12882-026-05022-9 (PMC13343869; doi:10.1186/s12882-026-05022-9)
Supplement: Supplementary file 1 — Supplementary Material 1 [file 12882_2026_5022_MOESM1_ESM.docx]

Supplementary Table 1. Baseline characteristics of participants according to serum pentosidine levels using eGFR calculated by the CKD-EPI equation.

|  | Total (N=813) | High Pentosidine (N=407) | Low Pentosidine (N=406) | P-value |
| --- | --- | --- | --- | --- |
| Age (years) | 63.9 (9.4) | 66.1 (7.9) | 61.7 (10.3) | <0.01 |
| Male, n (%) | 371 (45.6) | 178 (43.7) | 193 (47.5) | 0.29 |
| BMI (kg/m²) | 22.7 (3.2) | 22.1 (3.0) | 23.4 (3.3) | <0.01 |
| Waist circumference (cm) | 82.7 (9.1) | 80.9 (8.6) | 84.7 (9.2) | <0.01 |
| Systolic BP (mmHg) | 127.1 (17.0) | 126.4 (17.1) | 127.9 (16.9) | 0.22 |
| Diastolic BP (mmHg) | 74.3 (11.0) | 73.5 (10.5) | 75.2 (11.4) | 0.022 |
| eGFR (mL/min/1.73 m²) | 95.5 (10.7) | 92.9 (10.5) | 98.3 (10.2) | <0.01 |
| eGFR category, n (%) |  |  |  | <0.01 |
| eGFR ≥90 mL/min/1.73 m² (%) | 635 (78.1) | 294 (72.2) | 341 (84.0) |  |
| eGFR 60–89 mL/min/1.73 m² (%) | 178 (21.9) | 113 (27.8) | 65 (16.0) |  |
| eGFR <60 mL/min/1.73 m² (%) | 0 (0) | 0 (0) | 0 (0) |  |
| Urinary ACR (mg/g creatinine) | 3.61 (2.01–6.90) | 3.87 (2.19–7.45) | 3.41 (1.79–6.53) | 0.66 |
| Estimated salt intake (g/day) | 9.0 (2.0) | 9.0 (2.0) | 8.9 (2.0) | 0.29 |
| Estimated potassium intake (mg/day) | 3247 (784) | 3275 (781) | 3218 (787) | 0.29 |
| High-sensitivity CRP (mg/dL) | 0.040 (0.019–0.091) | 0.031 (0.015–0.073) | 0.049 (0.024–0.102) | <0.01 |
| Fasting glucose (mg/dL) | 97.7 (17.3) | 97.0 (14.5) | 98.5 (19.7) | 0.25 |
| HbA1c (NGSP, %) | 5.68 (0.58) | 5.68 (0.53) | 5.68 (0.63) | 0.96 |
| TG (mg/dL) | 96 (71–136) | 84 (66–118.5) | 107 (77–156) | <0.01 |
| LDL-C (mg/dL) | 134.0 (33.2) | 133.1 (33.9) | 135.0 (32.5) | 0.40 |
| HDL-C (mg/dL) | 68.7 (17.7) | 72.6 (18.0) | 64.8 (16.5) | <0.01 |
| Pentosidine (μg/mL) | 0.018 (0.014–0.022) | 0.026 (0.023–0.028) | 0.016 (0.013–0.019) | <0.01 |
| CML (μg/mL) | 4.02 (0.54) | 4.03 (0.52) | 4.01 (0.55) | 0.74 |

Values are presented as mean (standard deviation), median (interquartile range), or number (percentage), as appropriate.

Comparisons between groups were performed using the Student’s t-test for normally distributed continuous variables, the Mann–Whitney U test for non-normally distributed continuous variables, and the chi-squared test for categorical variables.

eGFR was calculated using the CKD-EPI 2021 equation.

Abbreviations: BMI, body mass index; BP, blood pressure; eGFR, estimated glomerular filtration rate; Cr, creatinine; ACR, albumin-to-Cr ratio; CRP, C-reactive protein; HbA1c, hemoglobin A1c; NGSP, National Glycohemoglobin Standardization Program; TG, triglycerides; LDL-C, low-density lipoprotein cholesterol; HDL-C, high-density lipoprotein cholesterol; CML, Nε-(carboxymethyl)lysine.

Supplementary Table 2. Factors associated with serum pentosidine levels: logistic and linear regression analyses using the CKD-EPI equation

|  | Univariable logistic regression | | | Multivariable logistic regression | | | Multivariable linear regression | | |
| --- | --- | --- | --- | --- | --- | --- | --- | --- | --- |
|  | OR | 95% CI | P value | OR | 95% CI | P value | B | 95% CI | P value |
| Age (per 10 years) | 1.58 | 1.36–1.83 | <0.01 | 1.55 | 1.31–1.83 | <0.01 | 0.05 | 0.01 to 0.08 | <0.01 |
| Sex (male) | 0.86 | 0.65–1.13 | 0.28 | 1.11 | 0.82–1.52 | 0.50 | 0.02 | -0.04 to 0.07 | 0.59 |
| Obesity (BMI ≥25 kg/m²) | 0.48 | 0.34–0.69 | <0.01 | 0.58 | 0.39–0.86 | <0.01 | -0.09 | -0.16 to -0.03 | <0.01 |
| CKD stage (per category decrease) | 2.02 | 1.43–2.84 | <0.01 | 1.87 | 1.29–2.71 | <0.01 | — | — | — |
| eGFR (per 10 mL/min/1.73 m² decrease) | — | — | — | — | — | — | 0.08 | 0.05 to 0.11 | <0.01 |
| Urinary ACR (>75th percentile) | 1.18 | 0.86–1.63 | 0.30 | 1.07 | 0.76–1.50 | 0.70 | 0.01 | -0.05 to 0.06 | 0.85 |
| High-sensitivity CRP (>75th percentile) | 0.66 | 0.48–0.91 | 0.01 | 0.71 | 0.50–1.00 | 0.05 | -0.07 | -0.13 to -0.01 | 0.03 |
| Systolic blood pressure (per 10 mmHg increase) | 0.96 | 0.89–1.04 | 0.36 | 0.94 | 0.86–1.03 | 0.21 | -0.02 | -0.03 to -0.003 | 0.02 |
| HbA1c (NGSP) ≥ 6.5% | 0.88 | 0.49–1.56 | 0.65 | 0.96 | 0.73–1.25 | 0.74 | — | — | — |
| HbA1c (NGSP) (per 1 % increase) | — | — | — | — | — | — | -0.01 | -0.06 to 0.03 | 0.54 |
| TG > 150 mg/dL | 0.42 | 0.29–0.60 | <0.01 | 0.50 | 0.34–0.74 | <0.01 | -0.09 | -0.16 to -0.03 | <0.01 |
| HDL-C < 40 mg/dL | 2.19 | 0.88–5.44 | 0.09 | 1.14 | 0.40–3.21 | 0.81 | 0.08 | -0.08 to 0.24 | 0.32 |

Odds ratios (ORs) and 95% confidence intervals (CIs) were estimated using logistic regression models. Regression coefficients (B) and 95% CIs were estimated using linear regression models.

Age was modeled per 10-year increase. eGFR was calculated using the 2021 CKD-EPI equation. CKD stage was treated as an ordinal variable (per category decrease), and eGFR was additionally analyzed as a continuous variable (per 10 mL/min/1.73 m² decrease). Obesity was defined as body mass index ≥25 kg/m². High urinary ACR and high hs-CRP were defined as values above the 75th percentile. High triglyceride levels were defined as ≥150 mg/dL, low HDL-C as <40 mg/dL, and high HbA1c as ≥6.5%.

Abbreviations: OR, odds ratio; CI, confidence interval; BMI, body mass index; CKD, chronic kidney disease; eGFR, estimated glomerular filtration rate; ACR, albumin-to-creatinine ratio; CRP, C-reactive protein; HbA1c, hemoglobin A1c; NGSP, National Glycohemoglobin Standardization Program; TG, triglycerides; HDL-C, high-density lipoprotein cholesterol.

Supplementary Table 3. Factors associated with serum pentosidine levels: logistic and linear regression analyses in participants with eGFR ≥60 mL/min/1.73 m²

|  | Univariable logistic regression | | | Multivariable logistic regression | | | Multivariable linear regression | | |
| --- | --- | --- | --- | --- | --- | --- | --- | --- | --- |
|  | OR | 95% CI | P value | OR | 95% CI | P value | B | 95% CI | P value |
| Age (per 10 years) | 1.50 | 1.27–1.75 | <0.01 | 1.47 | 1.24–1.76 | <0.01 | 0.07 | 0.04 to 0.10 | <0.01 |
| Sex (male) | 0.79 | 0.58–1.07 | 0.13 | 1.04 | 0.74–1.46 | 0.82 | 0.01 | -0.05 to 0.06 | 0.74 |
| Obesity (BMI ≥25 kg/m²) | 0.46 | 0.31–0.69 | <0.01 | 0.58 | 0.37–0.90 | 0.02 | -0.11 | -0.18 to -0.04 | <0.01 |
| CKD stage (per category decrease) | 2.05 | 1.13–3.72 | 0.02 | 1.72 | 0.91–3.24 | 0.09 | — | — | — |
| eGFR (per 10 mL/min/1.73 m² decrease) | — | — | — | — | — | — | 0.04 | 0.00 to 0.07 | <0.01 |
| Urinary ACR (>75th percentile) | 1.10 | 0.77–1.56 | 0.61 | 1.02 | 0.70–1.48 | 0.92 | -0.00 | -0.06 to 0.06 | 0.96 |
| High-sensitivity CRP (>75th percentile) | 0.63 | 0.44–0.91 | 0.01 | 0.69 | 0.46–1.03 | 0.07 | -0.09 | -0.15 to -0.03 | <0.01 |
| Systolic blood pressure (per 10 mmHg increase) | 0.97 | 0.89–1.06 | 0.46 | 0.96 | 0.87–1.06 | 0.38 | -0.01 | -0.03 to 0.00 | 0.15 |
| HbA1c (NGSP) ≥ 6.5% | 0.76 | 0.40–1.45 | 0.40 | 0.89 | 0.44–1.79 | 0.74 | — | — | — |
| HbA1c (NGSP) (per 1 % increase) | — | — | — | — | — | — | -0.02 | -0.07 to 0.03 | 0.41 |
| TG > 150 mg/dL | 0.49 | 0.33–0.72 | <0.01 | 0.57 | 0.37–0.86 | <0.01 | -0.07 | -0.13 to -0.002 | 0.046 |
| HDL-C < 40 mg/dL | 2.43 | 0.77–7.72 | 0.13 | 1.18 | 0.33–4.20 | 0.80 | 0.10 | -0.08 to 0.29 | 0.26 |

Odds ratios (ORs) and 95% confidence intervals (CIs) were estimated using logistic regression models. Regression coefficients (B) and 95% CIs were estimated using linear regression models.

Age was modeled per 10-year increase. CKD stage was treated as an ordinal variable (per category decrease), and eGFR was additionally analyzed as a continuous variable (per 10 mL/min/1.73 m² decrease). Obesity was defined as body mass index ≥25 kg/m². High urinary ACR and high hs-CRP were defined as values above the 75th percentile. High triglyceride levels were defined as ≥150 mg/dL, low HDL-C as <40 mg/dL, and high HbA1c as ≥6.5%.

Analyses were restricted to participants with eGFR ≥60 mL/min/1.73 m², calculated using the equation established by the Japanese Society of Nephrology.

Abbreviations: OR, odds ratio; CI, confidence interval; BMI, body mass index; CKD, chronic kidney disease; eGFR, estimated glomerular filtration rate; ACR, albumin-to-creatinine ratio; CRP, C-reactive protein; HbA1c, hemoglobin A1c; NGSP, National Glycohemoglobin Standardization Program; TG, triglycerides; HDL-C, high-density lipoprotein cholesterol.
